# Usefulness of a multiparametric evaluation including global longitudinal strain for an early diagnosis of acute myocarditis

**DOI:** 10.1007/s10554-021-02299-9

**Published:** 2021-06-04

**Authors:** Anna Degiovanni, Maria Concetta Pastore, Enrico Guido Spinoni, Marta Focardi, Matteo Cameli, Giuseppe Patti

**Affiliations:** 1 Department of Thoracic, Heart and Vascular Diseases, Maggiore Della Carità Hospital, Novara, Italy; 2 Department of Translational Medicine, University of Eastern Piedmont, Via Solaroli 17, 28100 Novara, Italy; 3 Department of Medical Biotechnologies, Division of Cardiology, University of Siena, Siena, Italy

**Keywords:** Acute myocarditis, Echocardiography, Global longitudinal strain, Speckle tracking, Cardiac magnetic resonance

## Abstract

Cardiac magnetic resonance imaging (CMRI) represents the main imaging modality for diagnosing acute myocarditis. However, its limited availability could entail missing or delayed diagnosis. A reduction of left ventricular global longitudinal strain (LV GLS) by speckle tracking echocardiography (STE) correlates with amount of oedema in acute myocarditis and here may be early detected. Aim was to evaluate the diagnostic and prognostic role of 3-layers LV GLS in patients with acute myocarditis. Out of 122 patients with suspected acute myocarditis, a total of 86 consecutive patients with CMRI-confirmed acute myocarditis admitted in two Italian institutions were retrospectively screened. Exclusion criteria were met in 29 patients because of poor acoustic window or missing data. A total of 57 patients were then included. Clinical characteristics, laboratory examinations, transthoracic echocardiography data and STE parameters were collected early after hospitalization. In the study population, mean age was 38.8 ± 15.6 years, the prevalence of male gender was 90%. On admission, 22 patients (39%) had fever (body temperature > 37.5 °), mean white blood cell (WBC) count was 10.9 ± 1.7/10^3 and overall LV ejection fraction was 50.1% ± 11.2. An epicardial LV GLS < 18% was present in 74% of patients, and a model including at least one of LV GLS < 18% (absolute value), fever and WBC > 10.0/10^3 was able to identify all patients with CMRI-diagnosed acute myocarditis. An epicardial LV GLS < 15.3% (absolute value) at baseline significantly predicted the lack of myocarditis resolution during follow-up (AUC 0.76, 95% CI 0.58–0.93, *p* = 0.02). A multiparametric model including epicardial LV GLS, fever and elevated WBC count on admission could be useful for early diagnosing an acute myocarditis, especially when CMRI is not promptly available. Baseline epicardial LV GLS may also identify patients with less-likely myocarditis resolution.

**Supplementary Information:**

The online version contains supplementary material available at 10.1007/s10554-021-02299-9.

## Background

Acute myocarditis is an acute inflammatory disease involving the myocardium, diagnosed by established clinical, laboratory and histological parameters [1]. The diagnosis of acute myocarditis can be challenging, as requires the exclusion of various other cardiac disorders, in particular coronary artery disease or valve disease. Nowadays, endomyocardial biopsy still represents the gold standard for the diagnosis of acute myocarditis, but its use is extremely limited in clinical practice [2]. In acute myocarditis, cardiac magnetic resonance imaging (CMRI) offers the highest diagnostic accuracy and is considered the main non-invasive diagnostic imaging modality [1]. However, in several institutions all over the world CMRI is not available or cannot be performed in the early phase of the disease, with possible missing or delaying in diagnosis. On the other hand, transthoracic echocardiography (TTE) is widely available and in acute myocarditis may help in performing differential diagnosis with non-inflammatory cardiac diseases and monitoring patients for early changes of cardiac chamber size, ventricular function and concomitant pericardial effusion. Moreover, the use of TTE with left ventricular global longitudinal strain (LV GLS) by speckle tracking echocardiography (STE) is rapidly growing in clinical practice. An impairment in LV GLS has been early detected in patients with acute myocarditis, with the degree of such impairment being related to the amount of oedema [3]. Few studies have been published on the utilization of STE for a precocious diagnosis of acute myocarditis and therefore current evidence on the topic is limited [4, 5]. Furthermore, given the heterogeneous presentation modalities of acute myocarditis, an integration of STE with other parameters, such as clinical presentation and laboratory exams, in particular inflammatory markers, might improve the diagnostic process.

On this basis, we present here a multicentre, retrospective study on the diagnostic accuracy of STE for an early diagnosis of acute myocarditis, with a particular focus on a non-invasive, integrative model including LV GLS and clinical/inflammatory parameters. Aim was also to correlate baseline LVGLS with myocarditis resolution during follow-up.

## Methods

### Study population

Consecutive patients (*N* = 122) admitted in two Italian Institutions (Maggiore della Carità Hospital of Novara and Santa Maria alle Scotte Hospital of Siena) from January 2015 to January 2018 for a clinical suspect of acute myocarditis and without severe reduction of left ventricular (LV) function (LV ejection fraction, LV EF, > 35%) were included in this observational study. Clinical parameters, also including body temperature (BT), results of laboratory tests [white blood cell count (WBC), C-reactive protein (CRP), haemoglobin and Troponin I (Tn-I)] and electrocardiographic data upon admission were collected. For fever definition, we set the threshold of a body temperature > 37.5 °C. All patients underwent invasive coronary angiography to exclude an acute coronary syndrome and received a TTE < 24 h from hospitalization. CMRI was performed < 2 weeks from admission and the diagnosis of acute myocarditis was done following the Lake Louise criteria [6]:

1. Regional or global myocardial signal intensity increase in T2-weighted images
2. Increased global myocardial early gadolinium enhancement ratio between myocardium and skeletal muscle in gadolinium-enhanced T1-weighted images
3. At least one focal lesion with non-ischemic regional distribution in inversion recovery-prepared gadolinium-enhanced T1-weighted images (“late gadolinium enhancement”, LGE).

If **two or more** criteria were present, or **criterion 3** was satisfied, CMRI was consistent with myocyte injury caused by myocarditis.

**Fig. 1:**
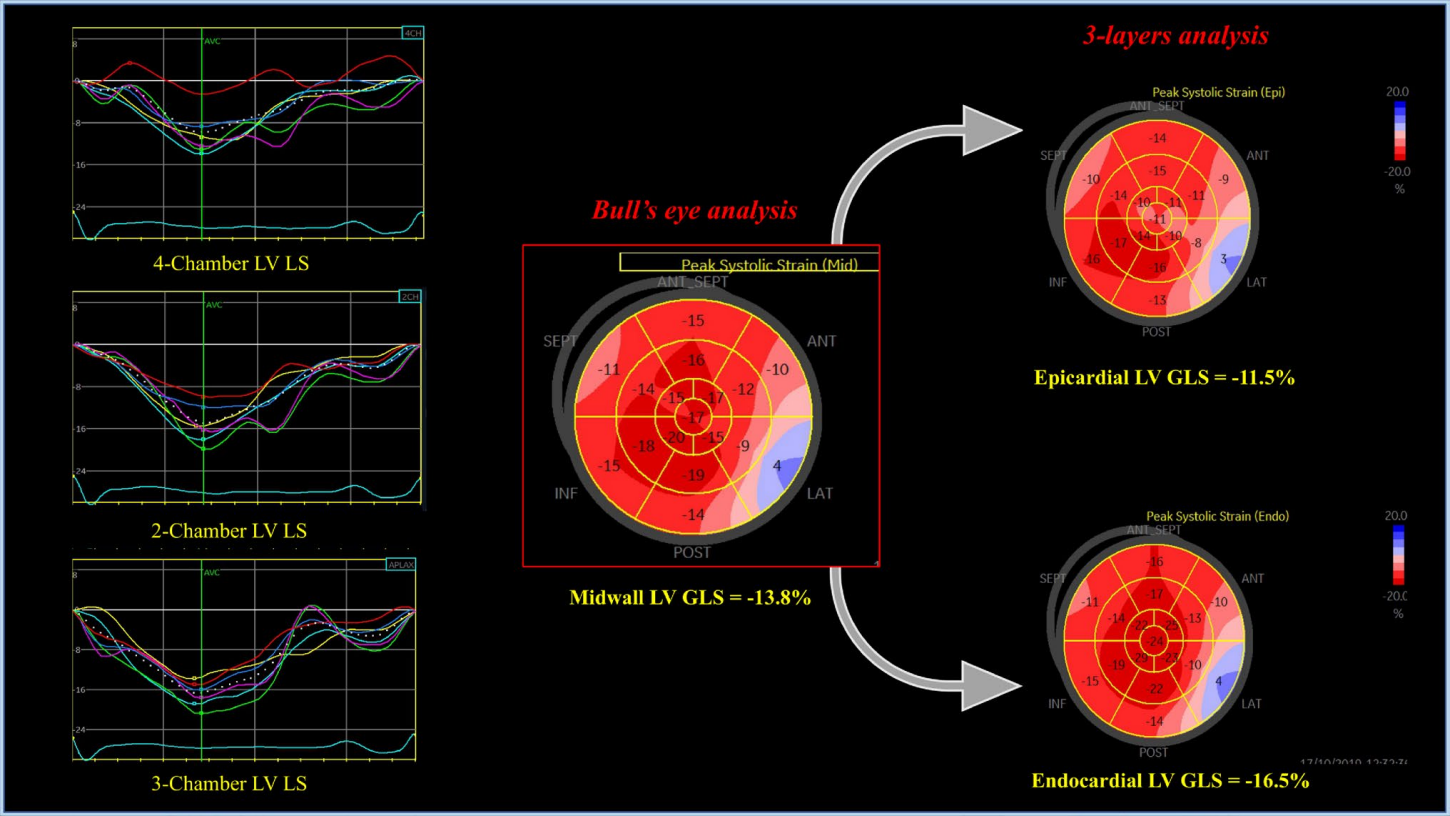
Measurement of LV GLS completed with bull’s eye and 3-layers analysis in a patient with acute myocarditis. Measures of LV strain are reported as absolute values. *GLS* Global longitudinal strain, *LV* Left ventricular

Exclusion criteria from the study were: poor acoustic window (*N* = 16); unconfirmed diagnosis of myocarditis (*N* = 36); missing data related to the index hospitalization (*N* = 13). Thus, out of the initial population of 122 patients, a total of 57 patients with CMRI-confirmed acute myocarditis were included in the analysis. The flow-chart leading to this final sample of the study population is illustrated in Fig. 1 of the Appendix. After a mean of 6.9 ± 3.2 months from the first CMRI exam, 31 patients underwent a new CMRI, in order to assess myocarditis resolution, which was defined as the complete absence of T2-weighted signal (myocardial oedema) [7].

### Echocardiographic evaluation

#### Basic echocardiography

TTE was performed by an experienced cardiologist using a high-definition echocardiography machine (*Vivid E9; GE Medical System, Northern, Norway*), equipped with a 2.5 MHz probe, in accordance with the American Society of Echocardiography (ASE)/European Association of Cardiovascular Imaging (EACVI) recommendations [8], with the subject in the left lateral recumbent position. LV diameters were measured in long-axis parasternal standard views. LV volumes, LV EF and left atrial (LA) volume and area were assessed using the biplane modified Simpson method from the apical 4- and 2-chamber views, as appropriate. Stroke volume (SV) was also measured as LV end-diastolic volume – LV end-systolic volume. SV and LA volumes were indexed to body surface area (BSA) to calculate left atrial volume index (LAVI) and SV index. M-mode measurement of the tricuspid annular plane systolic excursion (TAPSE) was also obtained from the apical 4-chamber view. Trans-mitral blood flow pattern was analyzed by pulsed wave Doppler, with the sample positioned at the tips of the mitral leaflets, recording maximum early diastolic (E) and late diastolic (A) velocities and calculating the E/A ratio. Tissue doppler imaging (TDI) was used to identify peak systolic (S’), early diastolic (E’) and late diastolic (A’) annular velocities, assessing “E’ avg” as the average value of mitral E’; then, E/E’avg ratio was calculated and used as index of the LV filling pressure. Valve dysfunctions were assessed using color-flow Doppler and quantified following the EACVI recommendations [9]. Tricuspid regurgitant velocity was measured by continuous wave Doppler and systolic pulmonary artery pressure (sPAP) was estimated as the sum of systolic trans-tricuspid pressure gradient and right atrial pressure derived from the diameter and collapsibility of the inferior vena cava.

#### Speckle tracking echocardiography

STE analysis was conducted on apical 2-, 3-, and 4- chamber view images obtained by 2D grey scale echocardiography with a stable electrocardiographic recording. STE evaluation was done by a unique experienced and independent examiner, who was not directly involved in the image acquisition and in the patient’s clinical management, using a commercially available semi-automated 2D strain calculation software (*EchoPac PC 113, GE, Milwaukee, Wisconsin*). A good visualization of the LV, from base to apex, and a reliable delineation of the endocardial border in each view was carefully obtained. Measurements from three consecutive heart cycles were recorded and averaged. The frame rate was set between 60 and 80 frames/sec. To measure LV strain, the endocardial border was manually traced in apical views, thus delineating a region of interest (ROI) including 6 segments for each view. The segmental tracking quality was analyzed, necessary manual adjustments of the ROI were performed and the longitudinal strain curves for each segment were then generated by the software. LV GLS was calculated as the average strain of all segments and reported as absolute value. Segments with inadequate tracking were excluded from the analysis. Moreover, using the “bull’s eye” function, the localization of segmental LV GLS reduction was assessed in a 17-segmental model, identifying 8 primary regions (anteroseptal, anterior, anterolateral, infero-septal, inferior, inferolateral, posterior, lateral). Finally, 3-layers analysis was used to assess LV strain distribution in the acute phase and investigate whether it was consistent with the typical early subepicardial LGE distribution by CMRI in acute myocarditis [1].

#### Three-layers analysis

We used the same software of conventional STE analysis (*EchoPac PC 113, GE, Milwaukee, Wisconsin*) for 3-layers analysis. This is performed after assessing LV longitudinal strain in the three apical views (4-chamber, 2-chamber and 3-chamber view) and calculating LV GLS, with a specific option of the software for 3-layers: this automatically calculated epicardial, mid-wall and endocardial LV GLS with relative curves (Fig. 1), which were recorded. Although not standardized, reference values for normality were derived from a recent study performed by Nagata et al. in healthy subjects [10].

### Cardiac magnetic resonance imaging

All patients underwent CMRI with the use of enhancing contrast. A superconductive 1.5 Tesla magnetic resonance scanner (*Intera Achieva; Philips, Netherlands*) with a cardiac phased-array coil and vectorcardiogram synchronization was utilized. Balanced turbo field echo (TFE) T2w short-axis and 2- and 4-chamber long-axis cine-sequences were obtained. A breath-hold balanced fast field echo sequence was used to evaluate wall motion and global LV function. Sequence parameters were: repetition time (TR) 3.8 ms, echo-time (TE) 1.92 ms, flip angle 60Åã, slice thickness 8 mm, matrix 192 × 512, field of view 500 mm, field of view (FOV) 50% and number of phases 30. In each patient, depending on LV volume, a total of 9–14 short-axis views and 2 long-axis views (4-chamber view and 2-chamber view, respectively) were acquired. Delayed enhancement (DE) images by a gradient echo inversion recovery sequence were obtained in all patients within 10–20 min after bolus injection of 0.2 mmol/kg of gadobutrol (*Gadovist., Schering, Germany*). A 2D-T1-weighted turbo-field-echo technique was used in the same short and long axis views. Sequence parameters were: TR 4.3 ms, TE 1.54 ms, flip angle 15Åã, slice thickness 10 mm, matrix 208 × 512 and field of view 350 mm, FOV 80%. Inversion time (200–320 ms) was optimized to a null signal from normal myocardium. LV volumes, mass and EF were measured using a previously validated software (EasyVision, version 4.0; Philips Medical Systems, Best, The Netherlands). The areas of LGE were assessed by visual approach with a scheme based on the transmural extent of LGE within each quartile (0–25%, 26–50%, 51–75% or > 75%). Diagnosis of acute myocarditis was confirmed if myocardial damage was evident by DE on the LV wall, with absence of involvement of the endocardial layer. Then, accordance between DE localization and LV GLS impairment shown by “bull’s eye” 17-segments model was registered.

### Statistical analysis

Continuous variables are indicated as mean ± standard deviation (SD) or as median [interquartile range (IQR)] in the case of non-normal distribution and were analyzed by t-test or Wilcoxon test, as appropriate. Data for categorical variables are reported as frequencies (percentage) and were analyzed by chi-square test. Receiver operating characteristic (ROC) curve analysis was used to test baseline clinical, laboratory and echocardiographic parameters and identify the best predictors of CMRI-confirmed myocarditis and their optimal cut-off values. These data were used to build a multiparametric model for the diagnosis of CMRI-confirmed acute myocarditis. Finally, the value of baseline clinical, laboratory and echocardiographic parameters for predicting CMRI-detected resolution of myocarditis was assessed.

Bland–Altman analysis was performed to evaluate inter-observer variability for speckle tracking measures on 20 randomly selected patients. Analyses were performed using the Statistical Package for Social Sciences software, release 20.0 (*SPSS*^*®*^*, Chicago, Illinois*); *p* values < 0.05 were considered statistically significant.

## Results

In the study population (*N* = 57), mean age was 38.8 ± 15.6 years and the prevalence of male gender was 90%. The diagnosis of acute myocarditis by CMRI was done at a mean of 8.14 ± 7.28 days from admission. Demographic/clinical characteristics are reported in Table 1, whereas laboratory parameters are presented in Table 2. On admission, 22 patients (39%) had fever, median CRP levels were 3.27 (IQR 1.015–6.17) mg/L, median WBC count was 11.2/10^3 (IQR 9130–11,650) and 25 patients (44%) had a WBC count > 10.0/10^3.

**Table 1:** Demographic/clinical characteristics at baseline (*N* = 57 patients)

Variable	Value
Age (years)	38.8 ± 15.6
Male	51 (90)
Body mass index (kg/m^2^)	24.8 ± 4.1
Arterial hypertension	14 (25)
Diabetes mellitus	4 (7)
Current smoking	22 (39)
Dyslipidemia	12 (21)
Electrocardiographic data	
Left bundle branch block	4 (7)
Right bundle branch block	6 (11)
Previous diagnosis of cardiomyopathy	
Hypertensive	7 (12)
Dilated	7 (12)
Ischemic	–
Fever (BT > 37.5 °C)	22 (37)
SBP (mmHg)	119.3 ± 23.7
DBP (mmHg)	74.8 ± 12.6

Data are presented as n. (%) or mean ± standard deviation

*BT* Body temperature, *DBP* Diastolic blood pressure, *SBP* Systolic blood pressure

**Table 2:** Laboratory parameters on admission

Variable	Value
Hb (g/dL)	14.2 (IQR 13.4–15.3)
WBC (n° × 10^3)	11,200 (IQR 9,130–11,650)
CRP (mg/L)	3.27 (IQR 1.015–6.17)
Serum creatinine (mg/dL)	0.9 (IQR 0.79–0.98)
Troponin I (ng/ml)	3.62 (IQR 0.835–8.65)

Data are presented as median (interquartile range)

*CRP* C-reactive protein, *Hb* Hemoglobin, *WBC* White blood cell count

TTE was performed at a mean of 18.0 ± 4.5 h after the hospitalization. In the overall population, TTE showed a mild reduction of LV systolic function (median LV EF = 54% (IQR 45–57) without impairment in diastolic function (E/A ratio 1.3 ± 0.6; E/e’avg ratio 7.6 ± 3.4). A complete description of echocardiographic parameters and STE data is reported in Tables 3 and 4. In particular, mean epicardial LV GLS (absolute value) was 14.5 ± 4.3%, mid-wall LV GLS (absolute value) = 16.5 ± 4.7% and endocardial LV GLS (absolute value) = 18.6 ± 5.0%. At 3-layers analysis, all patients showed a typical distribution of LV GLS with a gradual decrease from epicardial to endocardial layer. The majority of patients (*N* = 41; 73%) had at least two areas of regional LV strain reduction by bulls’ eye representation. An agreement in the localization between regional strain decrease and LGE at CMRI was present in 42 patients (74%).

**Table 3:** Standard echocardiography data and TDI parameters at baseline

Variable	Value
Standard echocardiography	
IVSd (mm)	10.6 ± 1.7
LV PWd (mm)	9.8 ± 1.1
LV EDD (mm)	50.6 ± 6.9
LV ESD (mm)	36.6 ± 9.3
LV EDV (mL/m^2^)	119.3 ± 43.4
LV ESV (mL/m^2^)	62.6 ± 41.9
LV EF (%)	54 (IQR 45–57)
E/A ratio	1.3 ± 0.6
LA volume (mL/m^2^)	50.1 ± 24.7
sPAP (mmHg)	27.5 ± 6.4
TAPSE (mm)	21.6 ± 3.8
MR > mild	8 (14)
AR > mild	1 (2)
LV SV (mL)	55.4 ± 15.4
LV SV index	30.3 ± 6.6
TDI	
E/E’_avg_ ratio	7.6 ± 3.4

Data are presented as *n*. (%), mean ± standard deviation or median (interquartile range)

*A* Late-diastolic wave by mitral annular pulsed-wave Doppler, *AR* Aortic regurgitation, *CMRI* Cardiac magnetic resonance imaging, *E* Early-diastolic wave by mitral annular pulsed-wave Doppler, *E’avg* Medium early mitral annular velocity by tissue doppler imaging (TDI), *EDD* End-diastolic diameter, *EDV* End-diastolic volume, *IQR* Interquartile range, *LA* Left atrium, *LVEF* Left ventricular ejection fraction, *ESD* End-systolic diameter, *ESV* End-systolic volume, *IVSd* Interventricular septum, measured in diastole, *MR* Mitral regurgitation, *Wd* Posterior wall measured in diastole, *sPAP* Systolic pulmonary artery pressure, *SV* stroke volume, *TAPSE* Tricuspid annular plane systolic excursion

**Table 4:** STE data and distribution of regional LV strain reduction at baseline

Variable	Value
STE	
Epicardial LV GLS^*^ (%)	14.5 ± 4.3
Mid-wall LV GLS^*^ (%)	16.5 ± 4.7
Endocardial LV GLS^*^ (%)	18.6 ± 5.0
Global TTP (ms)	359.0 ± 45.6
Agreement GLS-CMRI^#^	42 (74)
Distribution of regional strain reduction	
Antero-septal	22 patients (39%)
Anterior	21 patients (38%)
Antero-lateral	13 patients (23%)
Infero-septal	14 patients (25%)
Inferior	20 patients (36%)
Infero-lateral	21 patients (38%)
Posterior	11 patients (20%)
Lateral	23 patients (41%)

Data are presented as *n*. (%) or mean ± standard deviation

^*^Absolute value. ^**#**^agreement between regional strain decrease and localization of late gadolinium enhancement at CMRI

*CMRI* Cardiac magnetic resonance imaging, *GLS* Global longitudinal strain, *LV* Left ventricular, *STE* Speckle tracking echocardiography, *TTP* time to peak

**Fig. 2:**
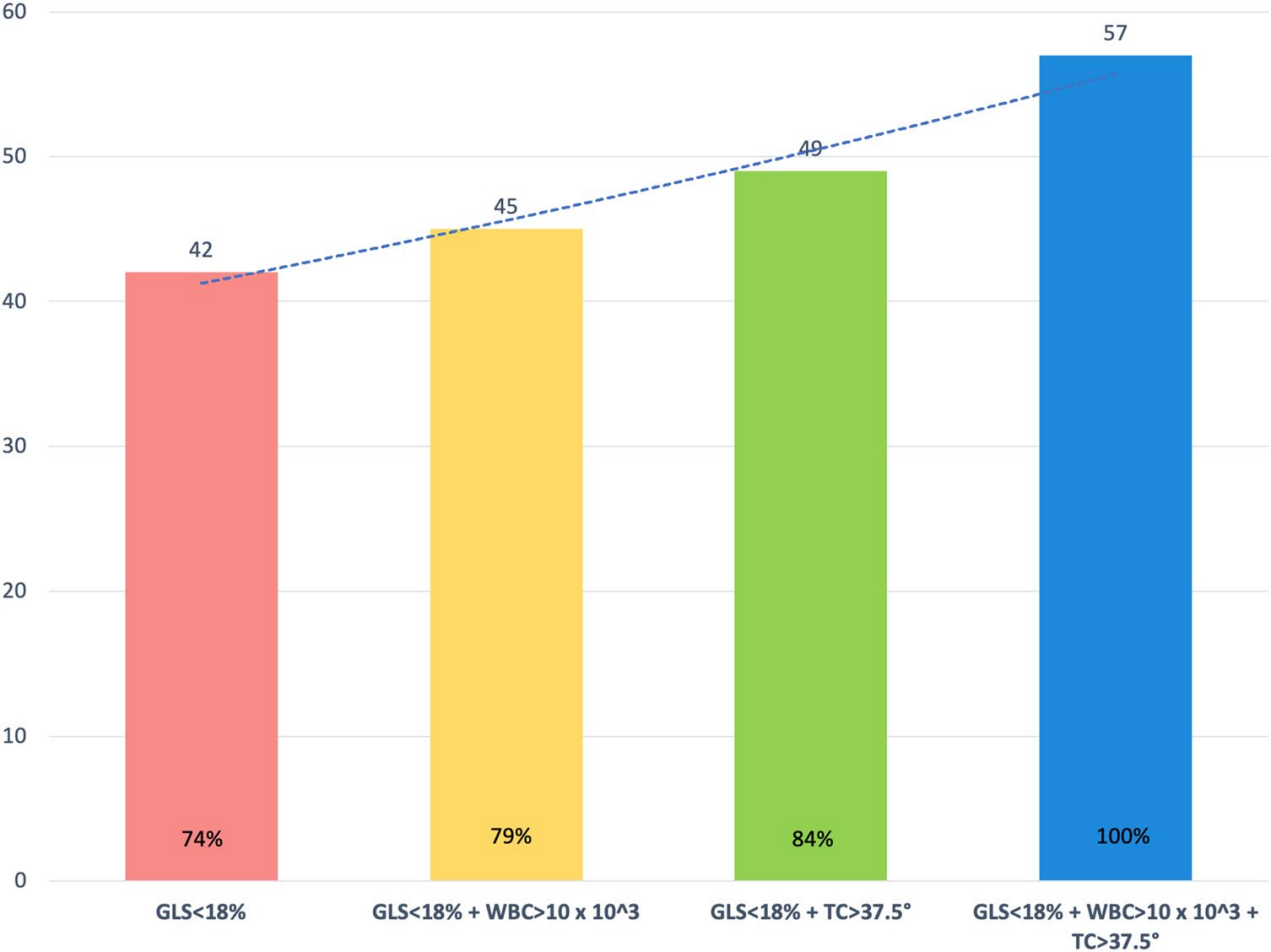
Incremental sensitivity of epicardial LV GLS alone, epicardial LV GLS plus WBC count, epicardial LV GLS plus fever and epicardial LV GLS plus WBC count or fever for the diagnosis of acute myocarditis. *BT* Body temperature, *GLS* Global longitudinal strain, *LV* Left ventricular, *WBC* White blood cell

**Fig. 3:**
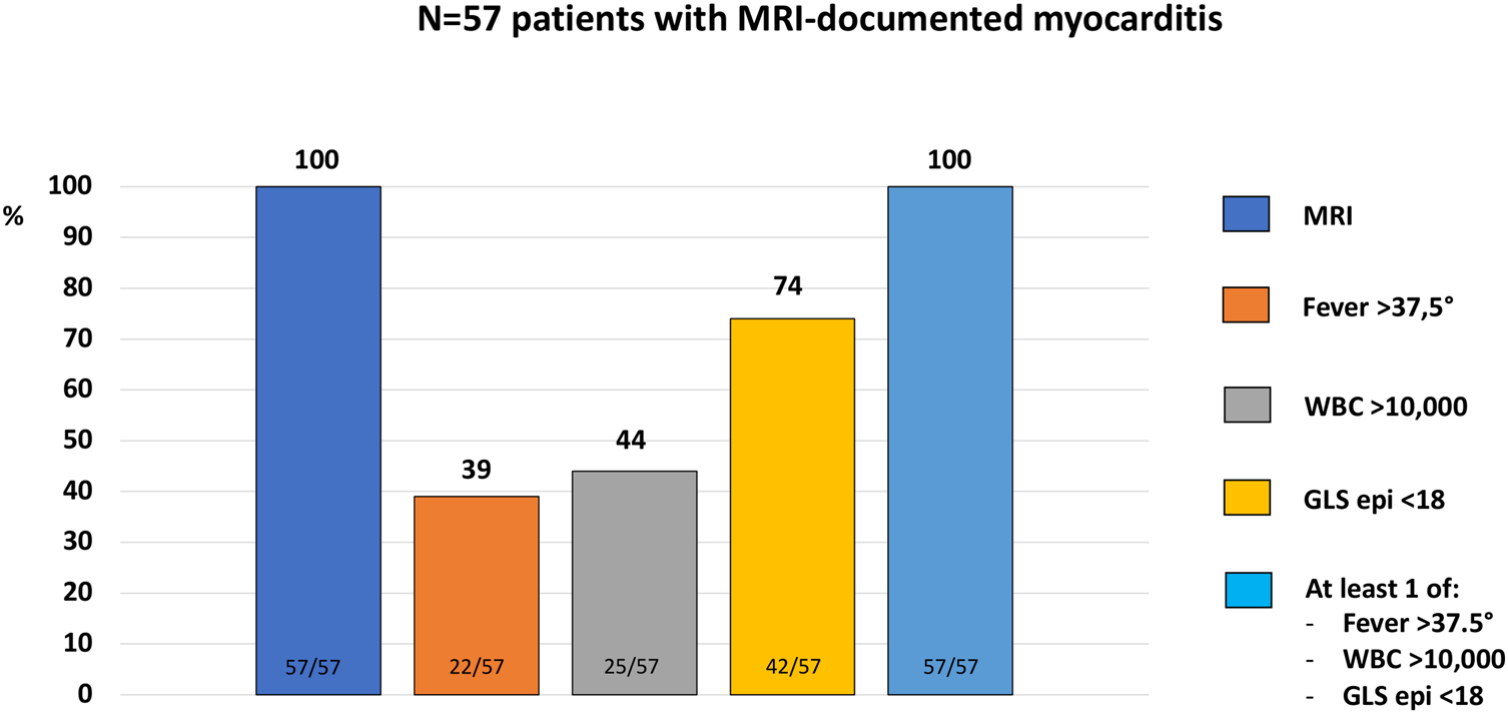
Multiparametric model for the diagnosis of acute myocarditis compared to CMRI, including fever, WBC count and epicardial LV GLS. *BT* Body temperature, *CMRI* Cardiac magnetic resonance imaging, *GLS* Global longitudinal strain, *LV* Left ventricular, *WBC* White blood cell

An epicardial LV GLS < 18% (absolute value) represented the optimal cut-off value for STE-CMRI localization agreement of acute myocarditis, with AUC 0.67 [95% CI 0.51–0.82], a sensitivity of 61% and a specificity of 70%. Notably, the localization agreement with CMRI was consistent for epicardial LV GLS and global LV GLS (61% and 57%, respectively; *p* = 0.66). We formulated a multiparametric model integrating STE data (epicardial LV GLS < 18%) and the following two parameters on admission: fever (BT > 37.5 °) and high WBC count (> 10.0/10^3). The presence of an epicardial LV GLS < 18% (absolute value) alone identified 74% of patients with CMRI-diagnosed acute myocarditis; the association of epicardial LV GLS < 18% (absolute value) and WBC > 10.0/10^3 identified 79% of patients with myocarditis, whereas the combination of LV GLS < 18% (absolute value) and BT > 37.5 ° identified 84% of patients (Fig. 2). Finally, the model including the presence of at least one of these three parameters was able to identify all patients (*n* = 57, 100%) with CMRI-diagnosed acute myocarditis (Figs. 2 and 3). AUC of mid-wall LV GLS for CMRI-diagnosed acute myocarditis was lower, but similar to the previously reported AUC of epicardial LV GLS (AUC 0.64, 95% CI 0.48–0.78; sensitivity = 63%, specificity = 62%).

As for prognostic purposes, an epicardial LV GLS < 15.3% (absolute value) was the best predictor of CMRI-detected resolution of myocarditis compared to either basic echocardiographic parameters and laboratory markers (Fig. 2 of the Appendix): AUC = 0.76 (95% CI 0.58–0.93; *p* = 0.02) vs AUC = 0.67 (95% CI 0.48–0.87; *p* = 0.1) for LV EF, AUC = 0.63 (95% CI 0.43–0.84; *p* = 0.2) for E/A, AUC = 0.58 (95% CI 0.37–0.80; *p* = 0.4) for LV stroke volume, AUC = 0.56 (95% CI 0.24–0.87; *p* = 0.07) for CRP values and AUC = 0.52 (95% CI 0.20–0.83; *p* = 0.9) for Tn-I. There was an inter-observer agreement of 98% for overall LV GLS measures.

## Discussion

This study indicates that an integration of clinical and laboratory parameters with STE of the LV, especially epicardial GLS, has a high consistency with the diagnosis of acute myocarditis provided by CMRI.

Patients included in our investigation had a CMRI-confirmed acute myocarditis, in whom an early assessment of STE upon admission demonstrated a proportional decrease of LV GLS from endocardial to epicardial layer. In the overall population, this LV GLS decrease paralleled a mild reduction of LV EF, without impairment of LV diastolic function. In particular, as a certain degree of LV GLS reduction was present in all patients, such parameter was used in a multiparametric model to evaluate its sensitivity for the diagnosis of acute myocarditis. An epicardial GLS < 18% (absolute value) had the highest agreement with CMRI findings, identifying alone acute myocarditis with a sensitivity of 74%. Notably, an integrated model including the presence of at least one of GLS < 18% (absolute value), BT > 37.5 ° and WBC > 10.0/10^3 was able to identify all patients with CMRI-diagnosed acute myocarditis. Thus, this assessment could be utilized to corroborate the diagnosis of acute myocarditis in patients where such condition is suspected, especially when CMRI is not promptly available. Importantly, to build the model we tested clinical features, as well as laboratory and echocardiographic variables, and we selected those parameters with the best discriminative performance for CMRI-diagnosed acute myocarditis. Other variables commonly used in clinical practice (for instance C-reactive protein levels) were not included, due to a poor discriminative power. However, our model does not represent a replacement of the diagnostic testing performed in clinical practice, e.g. coronary angiography for the differential diagnosis with acute coronary syndrome and CMRI to confirm the suspicion of acute myocarditis. Indeed, our model might allow to address the diagnostic suspicion in those centers were CMRI is not readily available.

STE has the great advantage to provide a sensitive, non-invasive and quick diagnostic evaluation in the acute phase of various cardiac diseases, since the images can be easily acquired bedside and immediately analyzed offline on a dedicated workstation. Importantly, in our study a limited proportion of patients (13%) were excluded because of a poor acoustic window precluding an adequate acquisition of STE data. Notably, this advanced echocardiographic technique is currently available in the large majority of institutions. After the publication of multicenter data and a consensus document by ASE/EACVI aimed to standardize its use and reference values overcoming vendor-dependency [11–14], it has been fully integrated into clinical practice. Previous investigations showed a significant correlation between STE and amount of myocardial oedema at CMRI in acute myocarditis [3]. At 3-layers analysis, such correlation with CMRI was prevalent for epicardial and endocardial LV GLS, and was not observed for other laboratory and echocardiographic indices, such as creatine-kinase MB, Tn, LV EF and wall motion score index.^3^ We also performed a 3-layers STE analysis to evaluate if GLS would follow the typical distribution of myocardial damage in the early phases of acute myocarditis, i.e. characterized by an epicardial involvement first and then mid-myocardial expansion in the following days. In fact, in the present study, LV GLS by STE outlined the typical epicardial-endocardial pattern of acute myocarditis, in agreement with CMRI findings on LGE distribution [6]. Moreover, LV GLS outperformed conventional echocardiographic parameters (e.g. LV ejection fraction; LV volumes) in detecting CMRI-confirmed acute myocarditis, as already previously described in other investigations [3, 15]. Thus, STE may represent a sensitive tool to identify areas of inflammatory myocardial involvement and to provide a differential diagnosis between acute myocarditis and coronary artery disease, where a typical endocardial-epicardial pattern is present. In our multiparametric model, variables commonly available in clinical practice were considered in addition to STE, in particular fever and WBC count. The wide reproducibility of this model offers the opportunity to rapidly diagnose an acute myocarditis in the initial phases of the disease (i.e. when in several centers CMRI cannot be promptly performed) and therefore to early initiate appropriate treatments. Importantly, LV GLS may represent a sensitive parameter able to detect subtle myocardial abnormalities also in patients with acute myocarditis and normal or subnormal EF. Overall, epicardial LV GLS < 18% may be considered a quite-sensitive diagnostic tool for mild cases of acute myocarditis in the hands of the experienced operators. However, the diagnosis should always be confirmed without any further delay by CMRI.

Finally, we performed a prognostic analysis, where epicardial LV GLS at baseline showed a moderate-to-high power to discriminate patients without myocarditis resolution at 6–8 months. Therefore, STE on admission could be also useful to stratify patients with poorer outcome during follow-up, who may benefit from a closer follow-up and “more aggressive” cardioprotective treatments.

Our study has limitations inherent to all observational and retrospective studies, mainly the risk of unmeasured residual confounding. Data collection on medical history and comorbidities was mainly based upon patient’s report, being therefore potentially biased. Acute myocarditis was diagnosed by CMRI, but we had no confirmatory data by endomyocardial biopsy and/or myocardial autopsy, still representing the gold standard for the diagnosis. Moreover, due to the retrospective nature of the study, we were not able to perform CMRI analysis by the newest technology for the assessment of myocardial fibrosis (i.e. T1 and T2 mapping; extracellular volume; black blood inversion recovery late gadolinium enhancement; tagging; feature tracking). Finally, the absence of control groups with nearly-missed Lake-Louise criteria or other diagnoses (such as acute pericarditis, acute coronary syndrome, healthy subjects) may have hampered the specificity of our analysis and may limit the interpretation of our findings.

In addition, LV GLS is reduced in a large spectrum of heart diseases (e.g. valve diseases, heart failure, LV hypertrophy or coronary artery disease) and therefore it has a low specificity. However, an integrated evaluation with clinical and laboratory parameters increases STE specificity. Moreover, a conscious interpretation of STE data may provide relevant information: for example, a regional reduction of LV GLS without relationship with a specific coronary distribution can be found in acute myocarditis, as opposed to acute and chronic coronary artery disease, where a close correlation between regional impairment of LV GLS and coronary distribution is observed [16].

In conclusion, although derived in absence of the gold standard method for diagnosing an acute myocarditis (i.e. myocardial biopsy), our results suggest that LV STE, in particular a reduction of epicardial GLS, have a high agreement with CMRI findings in patients with acute myocarditis. The integration of GLS data, body temperature and WBC on admission may represent a sensitive tool for an early diagnosis of acute myocarditis, especially when CMRI is not readily available. Moreover, an impaired LV GLS at baseline may be a reliable predictor of failed myocarditis resolution at mid-term. Our findings should be interpreted with caution and are purely hypothesis-generating. Prospective investigations on larger samples are needed to definitely confirm the diagnostic power of our multiparametric model in patients with acute myocarditis, as well as to specifically establish the predictive role of STE on clinical outcome during follow-up of these patients.

## Supplementary Information

Below is the link to the electronic supplementary material.

Supplementary file1—Figure 1 of the Appendix. Flow-chart of study population selection. Out of the initial population of 122 patients, a total of 57 patients with confirmed acute myocarditis were included in the analysis. *CMRI*= Cardiac magnetic resonance imaging (TIFF 11722 kb)

Supplementary file2—Figure 2 of the Appendix. ROC curves comparing epicardial GLS of the left ventricle with basic echocardiography (Panel A) and laboratory parameters (Panel B) for the prediction of myocarditis resolution by CMRI during follow-up.
Adm= Admission; CRP= C-reactive protein; AUC= Area under the curve; E/A= Transmitral peak early/late diastolic velocity; EF= Ejection fraction; GLSmid= Mid-wall global longitudinal strain; LV= Left ventricular; ROC= Receiver operating characteristic; SV= Stroke volume (TIFF 8792 kb)
