# Supplementary figures and images for: Usefulness of a multiparametric evaluation including global longitudinal strain for an early diagnosis of acute myocarditis
Source: Int J Cardiovasc Imaging. 2021 Jun 4;37(11):3203–11. doi: 10.1007/s10554-021-02299-9 (PMC8557136; doi:10.1007/s10554-021-02299-9)

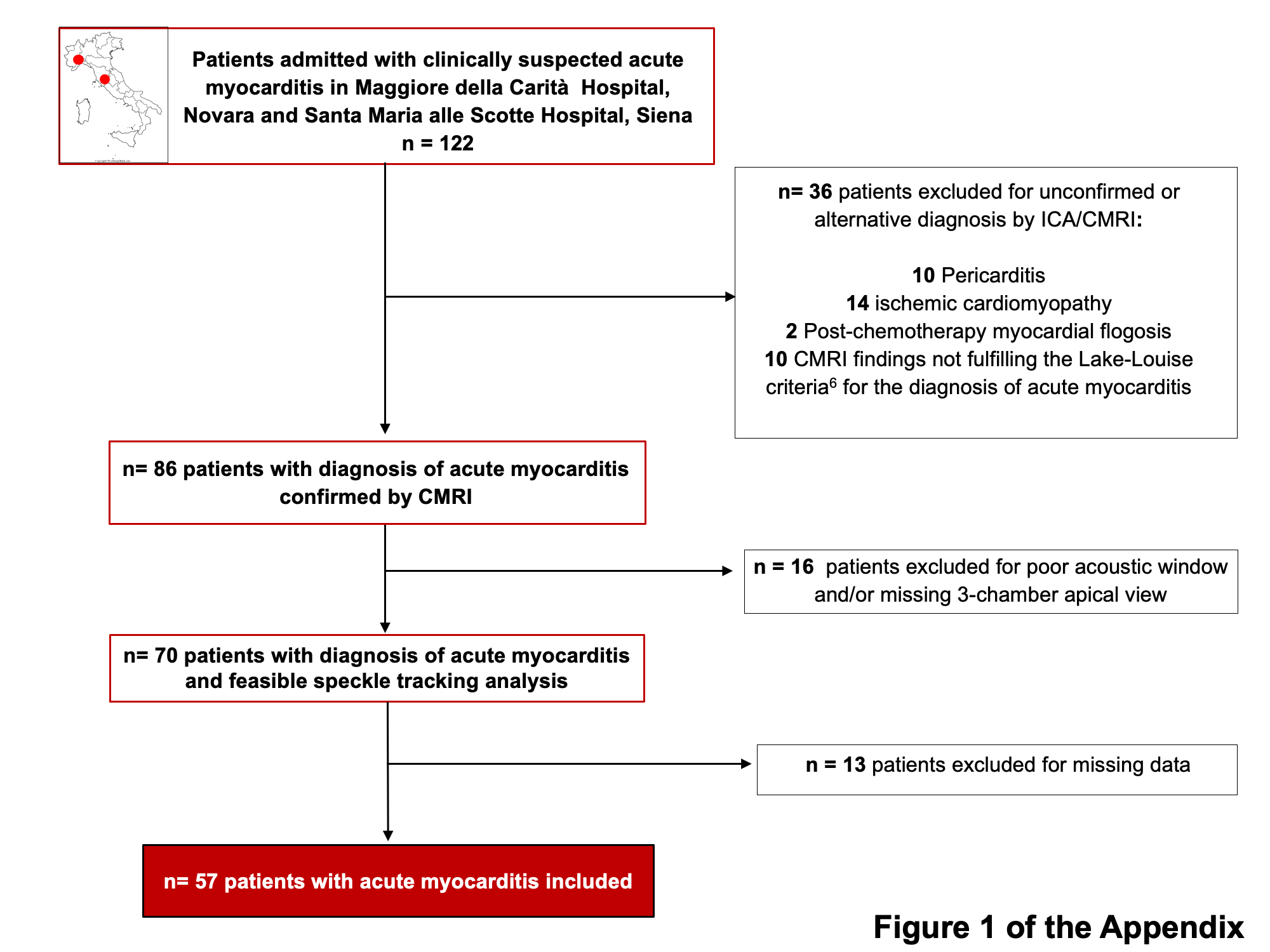

Supplement: Supplementary file 1 — Supplementary file1—Figure 1 of the Appendix. Flow-chart of study population selection. Out of the initial population of 122 patients, a total of 57 patients with confirmed acute myocarditis were included in the analysis. CMRI= Cardiac magnetic resonance imaging (TIFF 11722 kb) [file 10554_2021_2299_MOESM1_ESM.tiff]

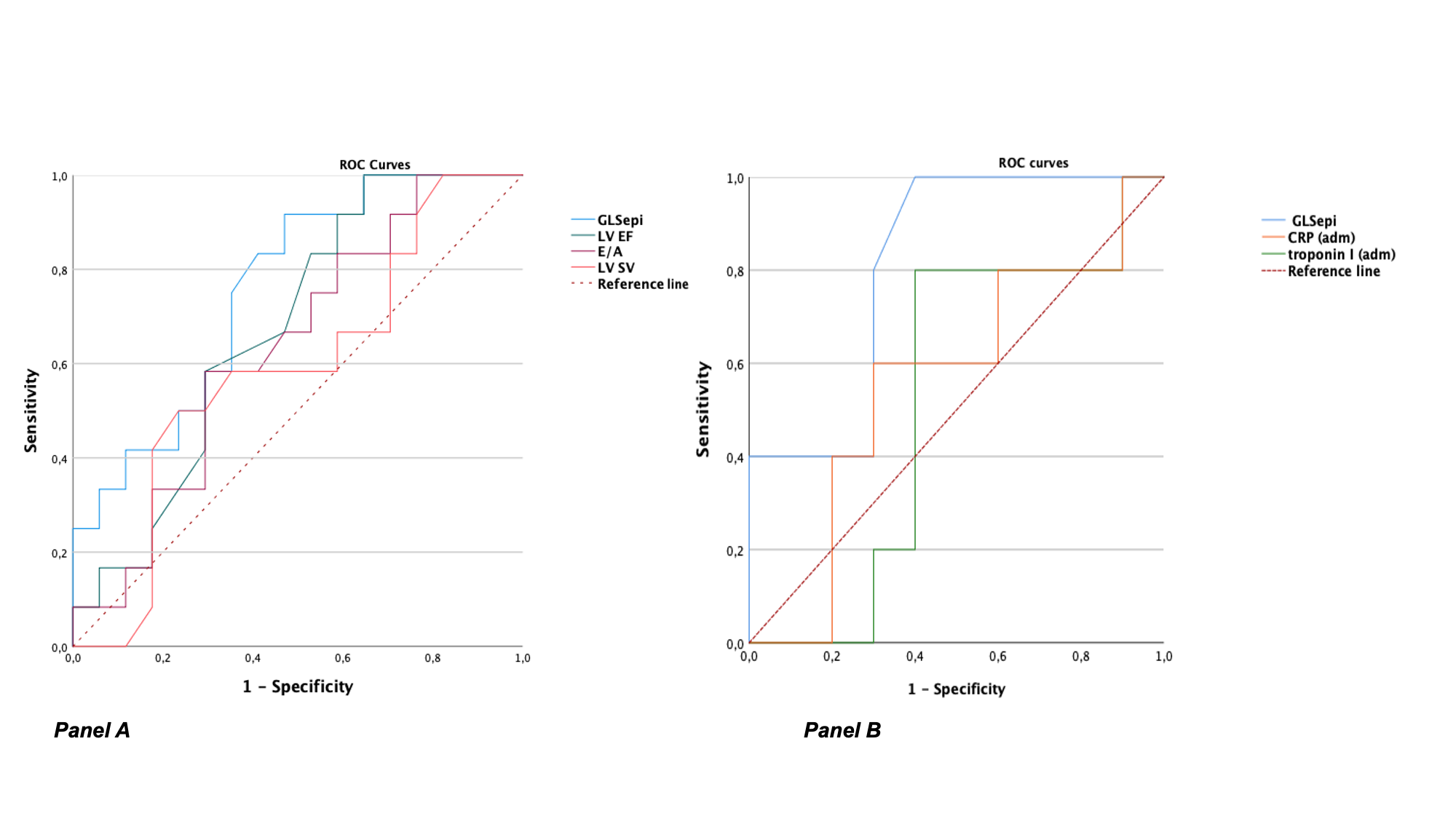

Supplement: Supplementary file 2 — Supplementary file2—Figure 2 of the Appendix. ROC curves comparing epicardial GLS of the left ventricle with basic echocardiography (Panel A) and laboratory parameters (Panel B) for the prediction of myocarditis resolution by CMRI during follow-up. Adm= Admission; CRP= C-reactive protein; AUC= Area under the curve; E/A= Transmitral peak early/late diastolic velocity; EF= Ejection fraction; GLSmid= Mid-wall global longitudinal strain; LV= Left ventricular; ROC= Receiver operating characteristic; SV= Stroke volume (TIFF 8792 kb) [file 10554_2021_2299_MOESM2_ESM.tiff]
